# Age-Related Weakness of Proximal Muscle Studied with Motor Cortical Mapping: A TMS Study

**DOI:** 10.1371/journal.pone.0089371

**Published:** 2014-02-21

**Authors:** Ela B. Plow, Nicole Varnerin, David A. Cunningham, Daniel Janini, Corin Bonnett, Alexandria Wyant, Juliet Hou, Vlodek Siemionow, Xiao-Feng Wang, Andre G. Machado, Guang H. Yue

**Affiliations:** 1 Department of Biomedical Engineering, Lerner Research Institute, Cleveland Clinic, Cleveland, Ohio, United States of America; 2 Department of Physical Medicine and Rehabilitation, Neurological Institute, Cleveland Clinic, Cleveland, Ohio, United States of America; 3 Quantitative Health Sciences, Lerner Research Institute, Cleveland Clinic, Cleveland, Ohio, United States of America; 4 Center for Neurological Restoration, Department of Neurosurgery, Neurological Institute, Cleveland Clinic, Cleveland, Ohio, United States of America; 5 Human Performance and Engineering Laboratory, Kessler Foundation Research Center, West Orange, New Jersey, United States of America; University of Toronto, Canada

## Abstract

Aging-related weakness is due in part to degeneration within the central nervous system. However, it is unknown how changes to the representation of corticospinal output in the primary motor cortex (M1) relate to such weakness. Transcranial magnetic stimulation (TMS) is a noninvasive method of cortical stimulation that can map representation of corticospinal output devoted to a muscle. Using TMS, we examined age-related alterations in maps devoted to biceps brachii muscle to determine whether they predicted its age-induced weakness. Forty-seven right-handed subjects participated: 20 young (22.6±0.90 years) and 27 old (74.96±1.35 years). We measured strength as force of elbow flexion and electromyographic activation of biceps brachii during maximum voluntary contraction. Mapping variables included: 1) center of gravity or weighted mean location of corticospinal output, 2) size of map, 3) volume or excitation of corticospinal output, and 4) response density or corticospinal excitation per unit area. Center of gravity was more anterior in old than in young (p<0.001), though there was no significant difference in strength between the age groups. Map size, volume, and response density showed no significant difference between groups. Regardless of age, center of gravity significantly predicted strength (β = −0.34, p = 0.005), while volume adjacent to the core of map predicted voluntary activation of biceps (β = 0.32, p = 0.008). Overall, the anterior shift of the map in older adults may reflect an adaptive change that allowed for the maintenance of strength. Laterally located center of gravity and higher excitation in the region adjacent to the core in weaker individuals could reflect compensatory recruitment of synergistic muscles. Thus, our study substantiates the role of M1 in adapting to aging-related weakness and subtending strength and muscle activation across age groups. Mapping from M1 may offer foundation for an examination of mechanisms that preserve strength in elderly.

## Introduction

Aging is associated with significant muscle weakness, ranging from 20% to 50% loss in strength [1]–[4]. Muscular atrophy contributes to this weakness [1], [5], [6]; however, degeneration within the central nervous system exaggerates the effect. Central neural degeneration with aging involves reduced gray matter volume [7], fewer motor cortical neurons [8], and decreased synaptic density [9], white matter integrity [10]–[12], neurotransmitter levels [13], spinal motoneuronal excitability [14]. Although the importance of central neural degeneration in aging-related weakness is well known [15], role of the primary motor cortex (M1) and corticospinal projections is relatively unclear.

Traditionally, M1 and its corticospinal output have been considered critical for dexterity [16] rather than force or muscle strength [17]. Single cell recordings in non-human primates however challenge this notion by showing that with increments in static torque, activity of corticospinal neurons increases linearly [18]. A similar relationship has been demonstrated in humans using Transcranial Magnetic Stimulation (TMS). By exciting M1 [19]–[21] via electromagnetic induction, TMS elicits motor evoked potentials (MEPs) in muscles, representing corticospinal excitation [22]–[24]. With increments of dynamic forces [25], [26], and with gains in muscle strength in training, TMS shows facilitation of corticospinal excitation [27]–[31].

It remains unknown, however, whether loss of muscle strength in aging is analogously related to reductions in corticospinal excitation. Evidence indicates that corticospinal excitation reduces with age [32]; intra-cortical and inter-hemispheric physiology becomes restrictive upon corticospinal output [33]–[38]. We have recently shown that intra-cortical and inter-hemispheric physiology relate to muscle strength in the aged [38]. However, such correlates of strength have been inferred only from one site in M1 [32], [39]–[43]. Since corticospinal output originates from several motor cortical areas [44], extrapolating from one site to infer system-wide reductions in corticospinal excitation may be contorted [45].

An alternate method that defines functional topography or representation of corticospinal output devoted to a muscle involves motor mapping. Mapping involves delivering TMS stimuli to various scalp sites along a coordinate system while MEPs are recorded from muscle-of-interest [24], [46]. Two recent studies have adopted mapping to define age-associated changes in corticospinal output for distal muscles of hand [47], [48]. This is not unlike other studies, a trend based on the significance of distal muscles for dexterity/skill [32], [39], [40]. We believe, however, that examining age-related adaptations that explain weakness, especially of the proximal large muscle groups, also carries significance. Even though loss of dexterity is the first to appear, weakness becomes prominent after 45 years of age [49]. Study of age-associated changes in corticospinal output for larger proximal muscles would be even more critical because their gross strength could compensate for failing dexterity in age, such as in stroke [50].

Therefore, in the present study, we examined motor maps devoted to biceps brachii muscle to understand whether altered representation of its corticospinal output explains age-induced weakness. The present study is an extension of our recent work [38], where we discussed how intra-cortical and inter-hemispheric physiology could explain age-induced weakness. In the present study, we compared motor maps of biceps across older and younger participants upon size of the map, location of weighted mean corticospinal excitation, and overall corticospinal excitation [46]. We theorized that motor maps would illustrate key age-related changes that would predict level of weakness. Study of motor maps in aging is clinically significant because in neurologic conditions that are associated with corticospinal and motoneuronal degeneration, such as stroke, spinal cord injury, or nerve injuries, motor maps reorganize in ways that is adaptive and at times maladaptive for weak muscles [51]–[53]. Since aging is analogously accompanied by corticospinal and motoneuronal degeneration [8], [39], [40], we theorized that motor maps in aging would reorganize to reflect age-induced weakness. Study of motor maps in relation to age-related weakness is also critical as it helps reinforce the role of M1 and corticospinal output in strength, an association classically emphasized for dexterity.

## Methods

### Ethics Statement

All subjects provided signed and written informed consent prior to participation. The Institutional Review Board of the Cleveland Clinic approved the experimental protocol.

### Subjects

Twenty young (mean ± s.e.) (22.6±0.90 years, 10 females) and 27 right-handed [54] older adults (74.96±1.35 years, 19 females) were enrolled. Young participants were recruited using advertisements around college campuses, while older participants were recruited from local community centers. Subjects had not been involved in systematic upper limb training for 5 years. Exclusion criteria included any confounding neurological or musculoskeletal condition affecting upper limbs, cognitive decline (tested using Mini Mental State Examination) [55] and established contraindication to TMS [56].

### Assessments

We chose to study the non-dominant biceps brachii because differences in corticospinal excitation between young and old are most accentuated on this side in right-handed participants [32].

1. **Elbow Flexion strength.** Elbow flexion force was measured on the left side. Subjects were seated in a chair with their left arm in slight abduction (∼10°), elbow flexion (90°) and forearm in neutral position. Upon verbal reinforcement, subjects generated maximal isometric elbow flexion force briefly (3–5 s) against a wrist cuff attached to a force transducer (JR3, Universal Force-Moment Sensor System, Woodland, CA) while feedback on the level of force was displayed on an oscilloscope (TDS 460 digitizing oscilloscope, Tektronix Inc., Beaverton, OR). They performed 5 trials separated by 45–60s of rest each. Surface electromyographic (EMG) signals were recorded from left biceps with bipolar electrodes (silver-silver chloride, 8mm diameter) positioned over the middle of the muscle belly.
2. **TMS recordings.** The motor map for non-dominant (left) biceps brachii was generated using TMS. Before the TMS session, the individual’s cranial landmarks were registered to a standard anatomical MRI template to assist stereotactic navigation for accurate application of TMS. TMS was applied using a figure-of-eight (70 mm) coil connected to a Magstim 200^2^ device (Magstim Co., Whitland, Dyfed, UK). The coil was placed tangentially on the scalp with the handle oriented backwards and laterally at 45° from mid-sagittal axis, while it was guided to target the anatomical region of the precentral gyrus in right M1, using frameless stereotaxy (Brainsight, Rogue Research Inc, Montreal, Canada). MEPs in biceps were recorded using surface EMG electrode set-up described above.

Using single-pulse TMS, we located the site which elicited MEPs of at least 50 µV (peak-to-peak amplitude) in biceps in 3 out of 5 trials at lowest TMS intensity; we denoted this site as the hotspot. The minimal intensity of TMS stimulation required to elicit such MEPs at the hotspot was termed the resting motor threshold (expressed as percentage of the maximum stimulator output).

The motor map was created at an intensity of 110% of the resting motor threshold [57], while subjects maintained biceps muscle at rest. Starting from the hotspot, scalp sites at incremental distances of 3 mm were targeted in eight radial directions over the right hemisphere on a three-dimensional reconstruction of the brain. We adopted this method because biceps is a proximal muscle, for which representation of corticospinal output can comprise fewer neurons spread over a variable region [58]. By using a method of sampling that not only maps in cardinal, but also in inter-cardinal directions from the hotspot, we aimed to sample at a higher resolution than contemporary work (generally 1cm in cardinal directions). During mapping, each site was stimulated with two successive TMS pulses separated by 4–6 seconds, based on methodology described previously [59], [60]. A site was deemed responsive only if it generated reproducible MEPs in resting left biceps on two consecutive trials [60]. Mapping was continued in each radial direction until two consecutive non-responsive sites were found. This method ensured that we were not limited to mapping within a grid of pre-specified points, enabling customization of mapping to each individual. Further, by mapping until even the smallest MEP (∼10 µV) could be discerned, we aimed to capture the full extent of representation of corticospinal output [61], [62]. The number of scalp sites in our study ranged from 14 to 96, with a mean±se of 47.93±2.59.

### Data Analysis

1. **Strength Measures.** Over 5 trials of maximum voluntary left elbow flexion, force signals were amplified (X 1000–3000), digitized at 200 Hz (1401 Plus, Cambridge Electronic Design, Ltd., Cambridge, UK), and recorded on computer for offline analysis. We computed mean force (in newtons, N). Voluntary EMG signals generated in left biceps during trials of maximum voluntary elbow flexion were amplified (X 500–5000), band-pass filtered (10Hz to 1 kHz) [model Cambridge Electronic Design 1902, Cambridge, UK], digitized (2,000 Hz), and full-wave rectified. EMG was analyzed in a 1s period at the time of maximal force during a trial. Over 5 trials, we defined the mean EMG (in millivolts, mV). The force (strength) and EMG data (voluntary activation) were analyzed offline using Spike2 (1401 Plus, Cambridge Electronic Design, Ltd., Cambridge, UK).
2. **Motor Map analysis.** MEPs from each scalp site were amplified, band-pass filtered (10Hz-2KHz) and digitized (4 kHz) (PowerLab 4/25T, ADD instruments, Salt Lake City, UT) and stored on a computer for offline analysis (Scope software version 4.0.8). The two non-rectified MEPs at each site were averaged. Their peak-to-peak amplitude was recorded and normalized to MEP_Maxima_, the maximum MEP (in mV) evoked in biceps muscle from any scalp site included in the motor map.
  - *Location of mean corticospinal excitation* , commonly known as center of gravity [57], [62], was expressed as MEP-weighted center. It illustrates the weighted-average location of representation of corticospinal output devoted to a muscle [62]–[65]. We described its medio-lateral (x) and antero-posterior (y) coordinates in relation to the nasion. The medio-lateral (x) coordinate was computed by multiplying the medio-lateral coordinate at each site by its normalized MEP and summing across all positions. The antero-posterior (y) coordinate was calculated using the same method. The equations are noted in 1,

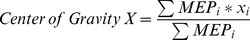
(1.1)

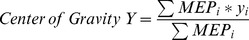
(1.2)where MEP_i_ is the average normalized amplitude of MEP at each responsive site and (x_i_, y_i_) represent the x and y coordinates of the site normalized to the nasion.

- *Map Size:* Extent of the representation of corticospinal output devoted to biceps or map size was described as count or the number of scalp sites eliciting MEPs in biceps [64].
- *Corticospinal Excitation* was calculated in two ways
  - *Overall excitation* was represented by normalized map volume, [57], [63] or the sum of normalized MEPs across all responsive scalp sites [63], [66]–[68] (equation 2).

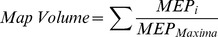
(2)Map volume was also extracted for four concentric sub-regions of the map. Sites that yielded MEPs between 0 to 25%, 25 to 50%, 50 to 75% and 75 to 100% of MEP_Maxima_ were categorized in different levels- level I, II, III and IV. To graphically represent map volume, we developed contour and three-dimensional plots [69].

- *Map Response Density:* Although map volume describes the total sum of *excitation*, it could be affected by map count. As in equation 2, map volume is defined by the sum of normalized MEPs at all sites. Thus, the variable is inherently linked to size. We therefore calculated a parameter, which we call response density that would quantify the spread of *excitation* irrespective of number of responsive sites constituting the map. Response density was defined as the average MEPs per unit area (equation 3). Biharmonic interpolation (Matlabv.R2009a) was used to 3D fit the data and these points were used in a double-integral formula to find the average MEP spread over the whole map. This average response was then normalized to map size for each individual. We computed the response density across the entire map, and then analyzed it for levels I, II, III and IV separately.
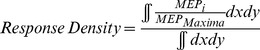
(3)

### Statistical Analyses

Statistical analysis was performed using Statistical Package for the Social Sciences (v18, SPSS Inc., Chicago, IL). Normality of all dependent variables was assessed with Kolmogorov-Smirnov tests and assessment of normality curves. Although we were aiming to understand age-related differences in strength, gender affects muscle strength and force as well. Females are generally weaker than males by up to 40% [70], [71]. Thus, comparisons of mean Force and mean EMG were conducted using 2-way Analysis of Variance (ANOVA), with age and gender as independent factors. The level of significance was set at α = 0.05. Variables related to motor map: size, center of gravity, map volume and response density were compared using two-tailed independent samples t-tests with appropriate corrections for multiple comparison test (Bonferroni method). Last, we employed a multiple regression analysis to estimate the relationship and predict the mean force of elbow flexion, and the mean EMG of biceps brachii, from variables of motor map, while accounting for the effect of age group and gender.

## Results

**Figure 1:**
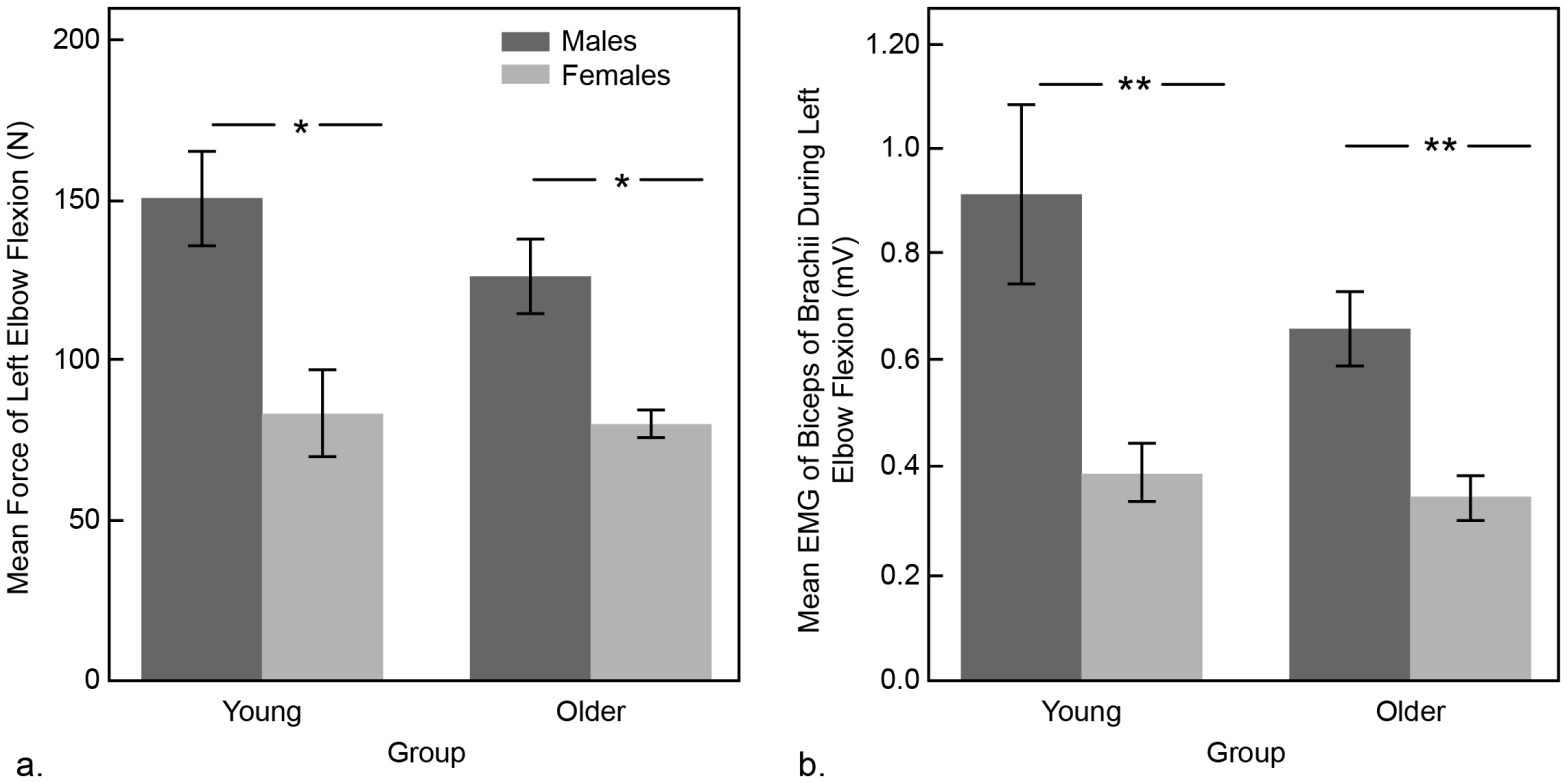
Effects of age and gender on strength. Findings of two-way analyses of variance exploring effect of age group and gender on (a) mean force of left elbow flexion and (b) mean EMG of biceps brachii. Older adults were not significantly weaker than young, but females were weaker than males in both age groups (* p≤0.05; ** p≤0.001).

All variables were normally distributed. Two-way ANOVA for mean force of elbow flexion showed that the main effect of gender was significant (F_1, 47_ = 28.49, p<0.001) but neither the main effect for age group nor its interaction with gender was significant. Females were approximately 42% weaker than males; while males generated 139.75±9.95N, females produced 81.28±5.38N of mean force during maximal elbow flexion. Comparisons of voluntary EMG activity of biceps brachii similarly revealed a main effect for gender (F_1, 47_ = 21.89, p<0.001), but no significant effect for age group or age group x gender interaction. In females, mean EMG of biceps brachii that was generated during maximal elbow flexion was less than half of what was generated in males (0.36±0.03mV versus 0.8±0.10mV). Females were weaker in both young and older age groups for mean force (*t_1,18_ = *3.33, p = 0.004 and *t_1,26_ = *4.41, p<0.001) and mean EMG of biceps (*t_1,18_ = *2.94, p = 0.014 and *t_1,26_ = *3.99, p<0.001) (Fig. 1a and 1b).

Resting motor threshold for young and older subjects was 66.55±3.86% and 64.82±2.52% of the maximum stimulator output. MEP_Maxima_ was 0.66±0.16 mV and 0.56±0.09 mV in younger and older individuals. Neither differed significantly between the two age groups.

**Figure 2:**
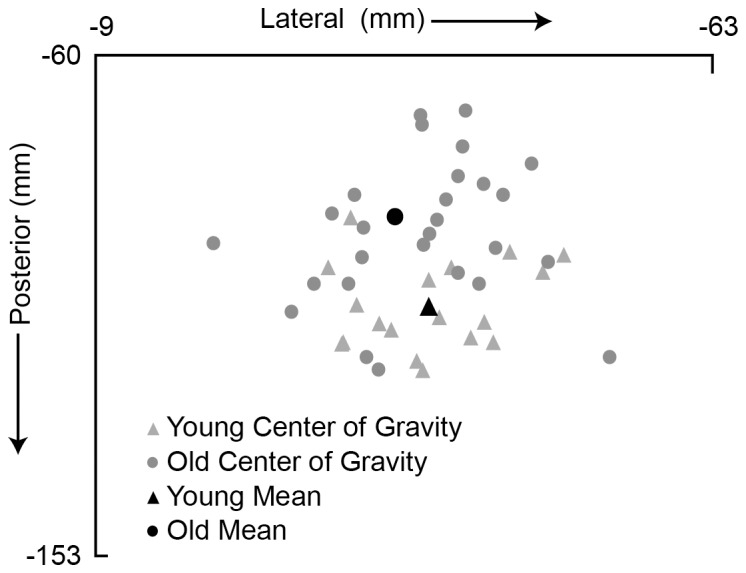
Center of gravity in young and old individuals. Location of center of gravity across all participants in both groups. As the averages demonstrate, center of gravity in older participants was more anterior.

The center of gravity of the map (Fig. 2) differed between the two age groups along the y-coordinate. Center of gravity _Y-Coordinate_ was significantly more posterior in the young versus the old (106.53±1.88 vs. 93.09±2.53 mm) (*t_0.05, 43_ = *3.88, p<0.001), whereas Center of gravity _X-Coordinate_ was comparable (−38.16±1.48mm in young versus −37.67±1.45mm in old).

**Figure 3:**
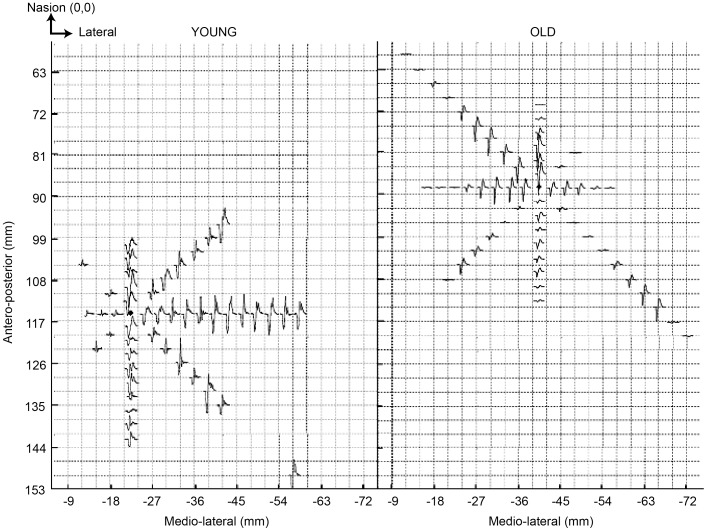
Representative motor maps in young and old individuals. Representative examples from a young and an older individual showing the size and spatial distribution of MEPs across the motor map of left biceps muscle in right hemisphere. The size (number of sites) did not vary significantly across groups (see also Fig. 4a). Note that map in the older individual is positioned anteriorly, which potentially explains why the center of gravity in older individuals was anterior too (Fig. 2).

**Figure 4:**
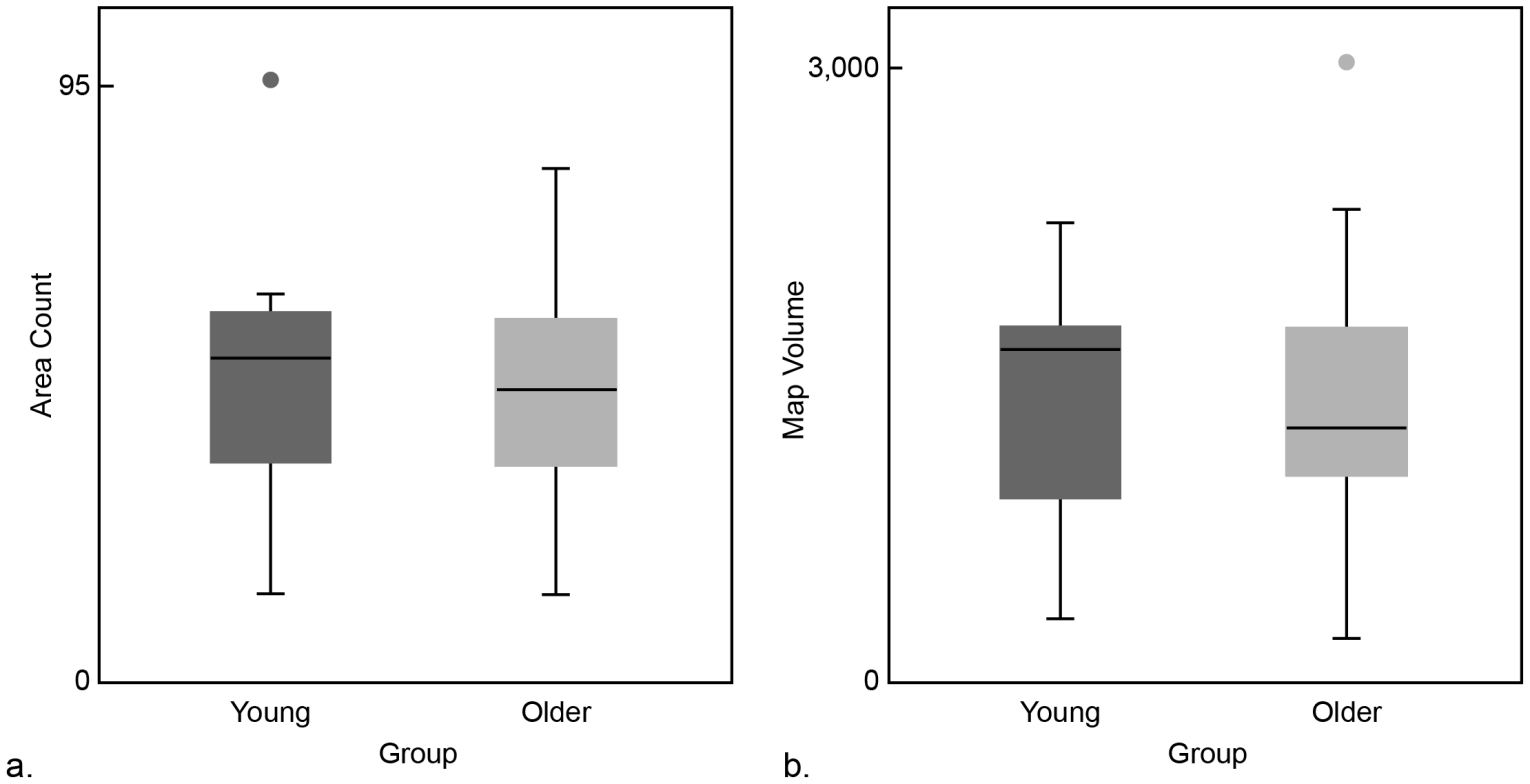
Map size and volume in young and old individuals. Differences between young and old subjects with respect to (a) map size or sites on scalp that are included in the motor map for left biceps muscle and (b) map volume. The groups did not differ significantly upon either measure although there is a seemingly higher count and volume in the younger group.

**Figure 5:**
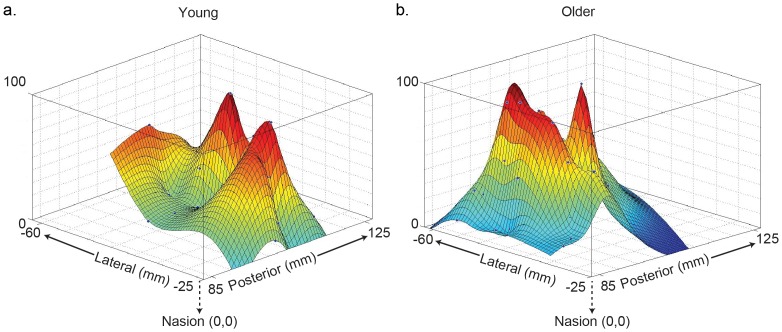
Age and corticospinal excitation. 5a,b shows 3-D function plots of the representative maps from a young and an old participant. These maps illustrate the spread of excitation across maps. Response densities did not significantly differ between age groups. The old subject has a response density of 25.39. The young subject has a response density of 21.63.

Figure 3 shows representative examples of motor maps from a young individual and an old individual. We have depicted the spatial distribution of MEPs across targeted loci comprising each map. The younger subject elicited MEPs in biceps from 52 loci while the older adult evoked responses from 42 (Fig. 3). The map count however was not different between the two age groups (young: 49.17±4.39; old: 45.85±3.34) (Fig. 4a). Comparisons of map volume (young: 1395.88±135.43 and old: 1339.73±128.61) (Fig. 4b) and its sub-levels I through IV did not vary between the young and old. Figure 5 illustrates the findings of spread of excitation, or the response density (Fig. 5a and b). The overall response density across the entire map was not different; it was 25.11±2.98 and 24.74±2.32 in young and older subjects, respectively. The two groups did not show a significant difference in any of the sub-levels, although response density in level IV tended to be lower in older versus young age group (84.72±0.39 vs. 83.43±0.47) (*t_0.05, 43_ = *1.94, p = 0.059), but did not meet the Bonferroni-corrected α level of significance set at 0.01. Representative examples can be found in Fig.5a and b.

Using the stepwise regression method, a significant model was observed in predicting left elbow flexion strength from characteristics of motor map (*F_2, 44_ = 20.01*, p<0.001). Adjusted R^2^ value signifying the proportion of the variance accounted by our model equaled 46.4%. Significant variables were center of gravity_X-Coordinate_ (β = −0.34, p = 0.005) and gender (β = 0.51, p<0.001). Age group was excluded from the regression because the 2-way ANOVA results showed that force was not different between age groups. Similarly, the stepwise regression method predicting voluntary activation (EMG) of biceps showed a significant model (*F_2, 44_ = 17.75*, p<0.001). Adjusted R^2^ value equaled 43.2%. Significant variables were map volume in level III (β = 0.32, p = 0.008) and gender (β = 0.63, p<0.001).

## Discussion

The purpose of the present study was to analyze age-related changes in motor map for biceps brachii, and assess if they explain age-induced differences in its strength. We have found that motor map of biceps muscle has a more anterior center of gravity in older adults, but it does not necessarily relate to age-induced decrement in strength of elbow flexion or biceps activation. When removing effect of age or gender, however, center of gravity explains differences in elbow flexion strength, while excitation of the map surrounding its core predicts voluntary muscle activation. Our study represents the first attempt at comparing aged versus young upon the entire representation of corticospinal output for a large, proximal upper limb muscle. This exploratory initiative has led to novel findings. We have shown adaptive reorganization that may help sustain corticospinal output for meaningful proximal muscle strength in the aged. Next, we have shown how representation of corticospinal output may be predictive of strength and muscle activation. These findings substantiate the role of M1 and corticospinal output in strength, offering correlates for study of weakness/de-conditioning, and strengthening.

The significant (∼13mm) age-related anterior shift of center of gravity in older adults cannot be explained by the threshold intensity of TMS or map size or corticospinal excitation because these did not vary between young and old. Instead, it may reflect adaptive reorganization in the aged. In patients with neural degeneration, such as in spinal cord injury [72] or stroke [73], shifts in center of gravity notably serve to increase motor output from weak muscles. Although aging is characterized by progressive, rather than acute degeneration, still 35% of corticomotor neurons are lost by age 50 [74]. To sustain useful muscle function, older adults may need to rely on anteriorly located areas that project substantial (∼60%) direct corticospinal neurons [75], [76]. Functional imaging identifies anterior activation in the supplementary [77] and premotor cortices [37] in the aged. Since neither elbow flexion strength nor biceps activation reduced with age in our study, i.e. performance of the aged remained comparable to the young [77], an anterior shift in locus of mean corticospinal excitation perhaps allowed for maintaining output in the aged.

The fact that medio-lateral coordinates of center of gravity explained elbow flexion strength, when removing effect of age and gender, is a new finding. It can be understood in the context of longitudinal changes in strength. Since gains in strength are accompanied by higher corticospinal excitation [27]–[30] for trained muscles [78]–[81], a relatively medial center of gravity in stronger individuals may represent higher excitation of prime elbow flexor- biceps brachii. On the other hand, a more lateral coordinate in weaker individuals may reflect greater reliance upon laterally represented synergists - wrist and finger flexors. We cautiously draw this inference however since EMG of wrist and finger flexor or brachioradialis was not acquired. Still, our position derives from deafferentation conditions [59], [82]. In such conditions, centers of gravity of typically remote muscles can become proximate owing to their co-contractions. These co-contractions serve as adaptations for weak muscles [69]. Future studies can employ longitudinal designs of strengthening, or compare stronger versus weaker adults upon topography of biceps and its synergists.

Map volume or excitation surrounding the core of the map significantly explained voluntary activation of biceps. That this effect was specifically observed not for the core but its surrounding region indicates that the differences in weak versus strong muscle activation relate to spread of excitation. Higher spread may be meaningful for weaker adults and may have been shaped by intra-cortical inhibition [83]. We have recently shown [38] that individuals who poorly activate biceps demonstrate lower intra-cortical inhibition. Lower inhibition may help spread excitation to recruit adjacent synergistic muscles. Again, without EMG of synergists, however, our projections are speculative.

Still, our present findings that map reorganization relates to muscle strength can find support in our recent findings where we implicate intra-cortical circuits [38]. In the same set of subjects, we have recently found that weaker individuals show lower intra-cortical inhibition, which may help them recruit neighboring muscles in increments in force or strength [29], [84], [85]. Since maps reorganize (shift) towards regions with lower intra-cortical inhibition [86], we believe that in the present report weaker individuals may have relied on intra-cortical physiology to spread excitation. In support, we have noted that area and excitability (map volume) were larger in those with lower intra-cortical inhibition (r = 0.29, p = 0.08, n = 36; r = 0.36, p = 0.03, n = 45) and lateral shift was noted in those with diffused excitability (low response density, r = 0.38, p = 0.009, n = 45) (data not shown regarding correlations with [38]). Weaker individuals may have shown lateral shift and spread of excitability to neighboring regions via reductions in lower intra-cortical inhibition. Even though without EMG of synergists, it is difficult to confirm whether map reorganization was truly representative of their recruitment, our findings here and their relation to our previous report of intra-cortical inhibition [38], preliminarily support our claim.

Overall, our study presents two novel directions in the study of age-associated changes in corticospinal excitation: motor map reorganization, and its relation to strength of a large, proximal upper limb muscle. Evidence thus far has almost invariably inferred age-associated corticospinal excitation from a single locus for distal muscles of wrist/hand [32], [39]–[43]. However, measuring excitation from a single locus misses a critical element - reorganization. Since reorganization defines disease-related shifts in excitation, particularly in stroke (review in [46]), understanding how age itself influences reorganization would help validly attribute changes in maps to disease vs. age. Study of corticospinal topography for a proximal muscle that has a role in gross motor strength rather than distal muscle that signifies dexterity is critical in the aged. Although distal muscles show greater and earlier reduction in corticospinal excitation than proximal in aging, strength of proximal muscles is essential to partially compensate for failing dexterity [50]. Our study lays the foundation for an exploration of mechanisms that preserve this important substrate of motor function.

Our study of maps irregularly compares with that of McGregor et al. [47], who show that maps in older adults have less excitation, and Bernard and Seidler, who note that they are spread extensively [48]. Owing to differences in methodology, comparing our study with others presents some challenge. Since we studied a proximal muscle that had not yet witnessed dramatic age-related weakness, we missed drastic age-related differences in excitation or map size. Had we examined topography of distal muscles in the same sample, our findings may have been relatable, a speculation supported by the fact that older subjects in present study were significantly weaker distally (grip force, data not shown). Also, since we used high-resolution mapping acquiring greater number of samples, relating our findings to those of others who use standard 1 cm resolution is challenging.

The present study and its methodology present limitations that warrant discussion. Compared to traditional mapping in a pre-determined (usually rectangular) grid, by mapping along radial directions we may have missed sites between orthogonal axes (see figure 3). We still believe there are some advantages unique to our technique. First, we collected data from ∼49 points in young and ∼45 in old, which is significantly greater than that tested with traditional rectangular grid maps (∼10 to 15) [48]. Second, the resolution of our map was three times better (3 mm) than that in standard mapping (usual 10 mm spacing). Our method may carry significance for proximal muscles as biceps that have fewer cortical sites [58], especially in stroke or other brain lesions where traditional 1 cm resolution maps are even smaller (∼4 to 5 sites) [60]. Further, with mapping at high resolution, even small changes in excitability can be meaningfully studied. We were able to explore spatial spread of excitability (response density) and detect changes within outer and inner layers of map (e.g. map volume and biceps’ activation). Another methodological limitation is that since several high-resolution sites were being collected, the number of trials at each was not adequate. Previous work has included two consecutive trials as here [60], but certainly TMS measures can suffer from poor trial-to-trial variability [87], [88]. In future, radial, high-resolution maps based on a pre-determined grid with sufficient measurements at each site would be ideal for mapping proximal muscle.

Finally, the lack of significant deficit in biceps strength may have resulted from a sampling issue. Older adults here were recruited from fitness facilities. Even though they had not engaged in upper limb training, they tended to be more active than those in the general population. Anterior shifts in center of gravity and lack of age-related weakness may be related to active lifestyles. Next, inclusion of different genders may have affected results when considering that subsets of older males and older females, and younger males and younger females were ultimately too small for powerful comparison. This limitation becomes clear when realizing that age group X gender interaction effect had p-values of 0.32 (observed power = 16.6%) and 0.27 (observed power = 19.7%) for strength and muscle activation. When we closely examine strength differences between older and younger males, the 2-tailed p-value for differences in their strength and muscle activation becomes 0.21 and 0.19. Therefore, we may have missed age-related differences in strength due to sampling issues and inclusion of smaller samples of both genders.

Although central neural degeneration has been implicated in muscle weakness in elderly, it is not known how changes in motor cortex explain changes in strength. Our study offers a novel exploratory perspective of such a relationship through study of maps of corticospinal excitation for a proximal muscle, strength of which is critical for function. We found both age-related and strength-related shifts in location and spread of its maps. Maps in older adults were anterior to those in young, constituting an adaptive shift, which potentially allowed for maintaining corticospinal excitation for sustaining strength. Strength-related shifts in maps offered weaker individuals an opportunity to spread corticospinal activation to recruit synergistic muscles. In elderly who have lost dexterity, the maintenance of strength in proximal muscles allows for their compensatory use to maintain overall motor function. While our study substantiates role of motor cortex in strength, still future studies could compare active, sedentary and frail older adults to assess if adaptive shifts in corticospinal excitation are an effect of active lifestyle or are vital to maintaining strength.

## References

[1] Doherty TJ (2003) Invited review: Aging and sarcopenia. J Appl Physiol 95: 1717–1727. 12970377 10.1152/japplphysiol.00347.2003

[2] Grabiner MD, Enoka RM (1995) Changes in movement capabilities with aging. Exerc Sport Sci Rev 23: 65–104. 7556361

[3] Hopp JF (1993) Effects of age and resistance training on skeletal muscle: a review. Phys Ther 73: 361–373. 8497511 10.1093/ptj/73.6.361

[4] Lindle RS, Metter EJ, Lynch NA, Fleg JL, Fozard JL, et al (1997) Age and gender comparisons of muscle strength in 654 women and men aged 20–93 yr. J Appl Physiol 83: 1581–1587. 9375323 10.1152/jappl.1997.83.5.1581

[5] Faulkner JA, Larkin LM, Claflin DR, Brooks SV (2007) Age-related changes in the structure and function of skeletal muscles. Clin Exp Pharmacol Physiol 34: 1091–1096. 17880359 10.1111/j.1440-1681.2007.04752.x

[6] Young A, Stokes M, Crowe M (1985) The size and strength of the quadriceps muscles of old and young men. Clin Physiol 5: 145–154. 3888498 10.1111/j.1475-097x.1985.tb00590.x

[7] Good CD, Johnsrude IS, Ashburner J, Henson RN, Friston KJ, et al (2001) A voxel-based morphometric study of ageing in 465 normal adult human brains. Neuroimage 14: 21–36. 11525331 10.1006/nimg.2001.0786

[8] Henderson G, Tomlinson BE, Gibson PH (1980) Cell counts in human cerebral cortex in normal adults throughout life using an image analysing computer. J Neurol Sci 46: 113–136. 7373341 10.1016/0022-510x(80)90048-9

[9] Haug H, Eggers R (1991) Morphometry of the human cortex cerebri and corpus striatum during aging. Neurobiol Aging 12: 336–338 discussion 352–335. 1961364 10.1016/0197-4580(91)90013-a

[10] Lindberg PG, Feydy A, Maier MA (2010) White matter organization in cervical spinal cord relates differently to age and control of grip force in healthy subjects. J Neurosci 30: 4102–4109. 20237280 10.1523/JNEUROSCI.5529-09.2010 PMC6632292

[11] Davis SW, Dennis NA, Buchler NG, White LE, Madden DJ, et al (2009) Assessing the effects of age on long white matter tracts using diffusion tensor tractography. Neuroimage 46: 530–541. 19385018 10.1016/j.neuroimage.2009.01.068 PMC2775533

[12] La Corte G, Wei Y, Chernyy N, Gluckman BJ, Schiff SJ (2013) Frequency dependence of behavioral modulation by hippocampal electrical stimulation. J Neurophysiol. 10.1152/jn.00523.2013 PMC3921403 24198322

[13] Gu Q (2002) Neuromodulatory transmitter systems in the cortex and their role in cortical plasticity. Neuroscience 111: 815–835. 12031406 10.1016/s0306-4522(02)00026-x

[14] Kido A, Tanaka N, Stein RB (2004) Spinal excitation and inhibition decrease as humans age. Can J Physiol Pharmacol 82: 238–248. 15181462 10.1139/y04-017

[15] Semmler JG, Kornatz KW, Meyer FG, Enoka RM (2006) Diminished task-related adjustments of common inputs to hand muscle motor neurons in older adults. Exp Brain Res 172: 507–518. 16489433 10.1007/s00221-006-0367-0

[16] Porter R, Lemon RN (1993) Corticospinal Function and Voluntary Movement. Oxford, UK: University Press.

[17] Remple MS, Bruneau RM, VandenBerg PM, Goertzen C, Kleim JA (2001) Sensitivity of cortical movement representations to motor experience: evidence that skill learning but not strength training induces cortical reorganization. Behav Brain Res 123: 133–141. 11399326 10.1016/s0166-4328(01)00199-1

[18] Cheney PD, Fetz EE (1980) Functional classes of primate corticomotoneuronal cells and their relation to active force. J Neurophysiol 44: 773–791. 6253605 10.1152/jn.1980.44.4.773

[19] Barker AT, Jalinous R, Freeston IL (1985) Non-invasive magnetic stimulation of human motor cortex. Lancet 1: 1106–1107. 2860322 10.1016/s0140-6736(85)92413-4

[20] Di Lazzaro V, Restuccia D, Oliviero A, Profice P, Ferrara L, et al (1998) Effects of voluntary contraction on descending volleys evoked by transcranial stimulation in conscious humans. J Physiol 508 (Pt 2): 625–633. 10.1111/j.1469-7793.1998.625bq.x PMC2230886 9508823

[21] Rothwell JC, Thompson PD, Day BL, Boyd S, Marsden CD (1991) Stimulation of the human motor cortex through the scalp. Experimental Physiology 76: 159–200. 2059424 10.1113/expphysiol.1991.sp003485

[22] Devanne H, Lavoie BA, Capaday C (1997) Input-output properties and gain changes in the human corticospinal pathway. Exp Brain Res 114: 329–338. 9166922 10.1007/pl00005641

[23] Ziemann U (2004) TMS and drugs. Clin Neurophysiol 115: 1717–1729. 15261850 10.1016/j.clinph.2004.03.006

[24] Siebner HR, Rothwell J (2003) Transcranial magnetic stimulation: new insights into representational cortical plasticity. Exp Brain Res 148: 1–16. 12478392 10.1007/s00221-002-1234-2

[25] Cros D, Soto O, Chiappa KH (2007) Transcranial magnetic stimulation during voluntary action: directional facilitation of outputs and relationships to force generation. Brain Res 1185: 103–116. 17961516 10.1016/j.brainres.2007.09.003

[26] Brouwer B, Sale MV, Nordstrom MA (2001) Asymmetry of motor cortex excitability during a simple motor task: relationships with handedness and manual performance. Exp Brain Res 138: 467–476. 11465745 10.1007/s002210100730

[27] Griffin L, Cafarelli E (2007) Transcranial magnetic stimulation during resistance training of the tibialis anterior muscle. J Electromyogr Kinesiol 17: 446–452. 16891123 10.1016/j.jelekin.2006.05.001

[28] Beck S, Taube W, Gruber M, Amtage F, Gollhofer A, et al (2007) Task-specific changes in motor evoked potentials of lower limb muscles after different training interventions. Brain Res 1179: 51–60. 17889840 10.1016/j.brainres.2007.08.048

[29] Weier AT, Pearce AJ, Kidgell DJ (2012) Strength training reduces intracortical inhibition. Acta Physiol (Oxf) 206: 109–119. 22642686 10.1111/j.1748-1716.2012.02454.x

[30] Lee M, Gandevia SC, Carroll TJ (2009) Short-term strength training does not change cortical voluntary activation. Med Sci Sports Exerc 41: 1452–1460. 19516155 10.1249/MSS.0b013e3181998837

[31] Liang N, Takahashi M, Ni Z, Yahagi S, Funase K, et al (2007) Effects of intermanual transfer induced by repetitive precision grip on input-output properties of untrained contralateral limb muscles. Exp Brain Res 182: 459–467. 17562034 10.1007/s00221-007-1004-2

[32] Sale MV, Semmler JG (2005) Age-related differences in corticospinal control during functional isometric contractions in left and right hands. J Appl Physiol 99: 1483–1493. 15947031 10.1152/japplphysiol.00371.2005

[33] Marneweck M, Loftus A, Hammond G (2011) Short-interval intracortical inhibition and manual dexterity in healthy aging. Neurosci Res 70: 408–414. 21536080 10.1016/j.neures.2011.04.004

[34] Peinemann A, Lehner C, Conrad B, Siebner HR (2001) Age-related decrease in paired-pulse intracortical inhibition in the human primary motor cortex. Neurosci Lett 313: 33–36. 11684333 10.1016/s0304-3940(01)02239-x

[35] Kossev AR, Schrader C, Dauper J, Dengler R, Rollnik JD (2002) Increased intracortical inhibition in middle-aged humans; a study using paired-pulse transcranial magnetic stimulation. Neurosci Lett 333: 83–86. 12419486 10.1016/s0304-3940(02)00986-2

[36] McGinley M, Hoffman RL, Russ DW, Thomas JS, Clark BC (2010) Older adults exhibit more intracortical inhibition and less intracortical facilitation than young adults. Exp Gerontol 45: 671–678. 20417265 10.1016/j.exger.2010.04.005 PMC2926152

[37] Talelli P, Ewas A, Waddingham W, Rothwell JC, Ward NS (2008) Neural correlates of age-related changes in cortical neurophysiology. Neuroimage 40: 1772–1781. 18329904 10.1016/j.neuroimage.2008.01.039 PMC3715371

[38] Plow EB, Cunningham D, Bonnett C, Gohar D, Bayram M, et al.. (2013) Neurophysiologic Correlates of Aging-Related Muscle Weakness. J Neurophysiol. 10.1152/jn.00205.2013 PMC3882769 24027104

[39] Pitcher JB, Ogston KM, Miles TS (2003) Age and sex differences in human motor cortex input-output characteristics. J Physiol 546: 605–613. 12527746 10.1113/jphysiol.2002.029454 PMC2342521

[40] Talelli P, Waddingham W, Ewas A, Rothwell JC, Ward NS (2008) The effect of age on task-related modulation of interhemispheric balance. Exp Brain Res 186: 59–66. 18040671 10.1007/s00221-007-1205-8 PMC2257995

[41] Wassermann EM (2002) Variation in the response to transcranial magnetic brain stimulation in the general population. Clin Neurophysiol 113: 1165–1171. 12088713 10.1016/s1388-2457(02)00144-x

[42] Oliviero A, Profice P, Tonali PA, Pilato F, Saturno E, et al (2006) Effects of aging on motor cortex excitability. Neurosci Res 55: 74–77. 16584795 10.1016/j.neures.2006.02.002

[43] Hortobagyi T, del Olmo MF, Rothwell JC (2006) Age reduces cortical reciprocal inhibition in humans. Exp Brain Res 171: 322–329. 16307241 10.1007/s00221-005-0274-9

[44] Morecraft RJ, Herrick JL, Stilwell-Morecraft KS, Louie JL, Schroeder CM, et al (2002) Localization of arm representation in the corona radiata and internal capsule in the non-human primate. Brain 125: 176–198. 11834603 10.1093/brain/awf011

[45] Reilly KT, Mercier C (2008) Cortical topography of human first dorsal interroseus during individuated and nonindividuated grip tasks. Hum Brain Mapp 29: 594–602. 17525982 10.1002/hbm.20421 PMC6870766

[46] Butler AJ, Wolf SL (2007) Putting the brain on the map: use of transcranial magnetic stimulation to assess and induce cortical plasticity of upper-extremity movement. Phys Ther 87: 719–736. 17429003 10.2522/ptj.20060274

[47] McGregor KM, Carpenter H, Kleim E, Sudhyadhom A, White KD, et al (2012) Motor map reliability and aging: a TMS/fMRI study. Exp Brain Res 219: 97–106. 22466408 10.1007/s00221-012-3070-3

[48] Bernard JA, Seidler RD (2012) Evidence for motor cortex dedifferentiation in older adults. Neurobiol Aging 33: 1890–1899. 21813213 10.1016/j.neurobiolaging.2011.06.021 PMC3391352

[49] Soer R, Brouwer S, Geertzen JH, van der Schans CP, Groothoff JW, et al (2012) Decline of functional capacity in healthy aging workers. Arch Phys Med Rehabil 93: 2326–2332. 22842482 10.1016/j.apmr.2012.07.009

[50] Canning CG, Ada L, O’Dwyer NJ (2000) Abnormal muscle activation characteristics associated with loss of dexterity after stroke. J Neurol Sci 176: 45–56. 10865092 10.1016/s0022-510x(00)00305-1

[51] Rijntjes M, Tegenthoff M, Liepert J, Leonhardt G, Kotterba S, et al (1997) Cortical reorganization in patients with facial palsy. Ann Neurol 41: 621–630. 9153524 10.1002/ana.410410511

[52] Liepert J, Bauder H, Wolfgang HR, Miltner WH, Taub E, et al (2000) Treatment-induced cortical reorganization after stroke in humans. Stroke 31: 1210–1216. 10835434 10.1161/01.str.31.6.1210

[53] Lotze M, Laubis-Herrmann U, Topka H (2006) Combination of TMS and fMRI reveals a specific pattern of reorganization in M1 in patients after complete spinal cord injury. Restor Neurol Neurosci 24: 97–107. 16720945

[54] Oldfield RC (1971) The assessment and analysis of handedness: the Edinburgh inventory. Neuropsychologia 9: 97–113. 5146491 10.1016/0028-3932(71)90067-4

[55] Folstein MF, Robins LN, Helzer JE (1983) The Mini-Mental State Examination. Arch Gen Psychiatry 40: 812. 6860082 10.1001/archpsyc.1983.01790060110016

[56] Rossi S, Hallett M, Rossini PM, Pascual-Leone A (2009) Safety, ethical considerations, and application guidelines for the use of transcranial magnetic stimulation in clinical practice and research. Clin Neurophysiol 120: 2008–2039. 19833552 10.1016/j.clinph.2009.08.016 PMC3260536

[57] Sawaki L, Butler AJ, Leng X, Wassenaar PA, Mohammad YM, et al (2008) Constraint-induced movement therapy results in increased motor map area in subjects 3 to 9 months after stroke. Neurorehabil Neural Repair 22: 505–513. 18780885 10.1177/1545968308317531 PMC3234527

[58] Brasil-Neto JP, McShane LM, Fuhr P, Hallett M, Cohen LG (1992) Topographic mapping of the human motor cortex with magnetic stimulation: factors affecting accuracy and reproducibility. Electroencephalogr Clin Neurophysiol 85: 9–16. 1371748 10.1016/0168-5597(92)90095-s

[59] Streletz LJ, Belevich JK, Jones SM, Bhushan A, Shah SH, et al (1995) Transcranial magnetic stimulation: cortical motor maps in acute spinal cord injury. Brain Topogr 7: 245–250. 7599023 10.1007/BF01202383

[60] Marconi B, Filippi GM, Koch G, Giacobbe V, Pecchioli C, et al (2011) Long-term effects on cortical excitability and motor recovery induced by repeated muscle vibration in chronic stroke patients. Neurorehabil Neural Repair 25: 48–60. 20834043 10.1177/1545968310376757

[61] Triggs WJ, Subramanium B, Rossi F (1999) Hand preference and transcranial magnetic stimulation asymmetry of cortical motor representation. Brain Res 835: 324–329. 10415389 10.1016/s0006-8993(99)01629-7

[62] Malcolm MP, Triggs WJ, Light KE, Shechtman O, Khandekar G, et al (2006) Reliability of motor cortex transcranial magnetic stimulation in four muscle representations. Clin Neurophysiol 117: 1037–1046. 16564206 10.1016/j.clinph.2006.02.005

[63] Tsao H, Danneels LA, Hodges PW (2011) ISSLS Prize Winner: Smudging the Motor Brain in Young Adults With Recurrent Low Back Pain. Spine (Phila Pa 1976) 36: 1721–1727. 21508892 10.1097/BRS.0b013e31821c4267

[64] Gagne M, Hetu S, Reilly KT, Mercier C (2011) The map is not the territory: Motor system reorganization in upper limb amputees. Hum Brain Mapp 32: 509–519. 21391244 10.1002/hbm.21038 PMC6870038

[65] Diekhoff S, Uludag K, Sparing R, Tittgemeyer M, Cavusoglu M, et al (2011) Functional localization in the human brain: Gradient-Echo, Spin-Echo, and arterial spin-labeling fMRI compared with neuronavigated TMS. Hum Brain Mapp 32: 341–357. 20533563 10.1002/hbm.21024 PMC6870385

[66] Karl A, Birbaumer N, Lutzenberger W, Cohen LG, Flor H (2001) Reorganization of motor and somatosensory cortex in upper extremity amputees with phantom limb pain. J Neurosci 21: 3609–3618. 11331390 10.1523/JNEUROSCI.21-10-03609.2001 PMC6762494

[67] Wolf SL, Butler AJ, Campana GI, Parris TA, Struys DM, et al (2004) Intra-subject reliability of parameters contributing to maps generated by transcranial magnetic stimulation in able-bodied adults. Clin Neurophysiol 115: 1740–1747. 15261852 10.1016/j.clinph.2004.02.027

[68] Hetu S, Gagne M, Reilly KT, Mercier C (2011) Short-term reliability of transcranial magnetic stimulation motor maps in upper limb amputees. J Clin Neurosci 18: 728–730. 21393001 10.1016/j.jocn.2010.09.011

[69] Tyc F, Boyadjian A (2011) Plasticity of motor cortex induced by coordination and training. Clin Neurophysiol 122: 153–162. 21168091 10.1016/j.clinph.2010.05.022

[70] Frontera WR, Hughes VA, Lutz KJ, Evans WJ (1991) A cross-sectional study of muscle strength and mass in 45- to 78-yr-old men and women. J Appl Physiol 71: 644–650. 1938738 10.1152/jappl.1991.71.2.644

[71] Kanehisa H, Ikegawa S, Fukunaga T (1994) Comparison of muscle cross-sectional area and strength between untrained women and men. Eur J Appl Physiol Occup Physiol 68: 148–154. 8194544 10.1007/BF00244028

[72] Freund P, Rothwell J, Craggs M, Thompson AJ, Bestmann S (2011) Corticomotor representation to a human forearm muscle changes following cervical spinal cord injury. Eur J Neurosci 34: 1839–1846. 22082003 10.1111/j.1460-9568.2011.07895.x

[73] Bastings EP, Greenberg JP, Good DC (2002) Hand motor recovery after stroke: a transcranial magnetic stimulation mapping study of motor output areas and their relation to functional status. Neurorehabil Neural Repair 16: 275–282. 12234089 10.1177/154596802401105207

[74] Eisen A, Entezari-Taher M, Stewart H (1996) Cortical projections to spinal motoneurons: changes with aging and amyotrophic lateral sclerosis. Neurology 46: 1396–1404. 8628489 10.1212/wnl.46.5.1396

[75] Dum RP, Strick PL (1991) The origin of corticospinal projections from the premotor areas in the frontal lobe. J Neurosci 11: 667–689. 1705965 10.1523/JNEUROSCI.11-03-00667.1991 PMC6575356

[76] He SQ, Dum RP, Strick PL (1993) Topographic organization of corticospinal projections from the frontal lobe: motor areas on the lateral surface of the hemisphere. J Neurosci 13: 952–980. 7680069 10.1523/JNEUROSCI.13-03-00952.1993 PMC6576595

[77] Hutchinson S, Kobayashi M, Horkan CM, Pascual-Leone A, Alexander MP, et al (2002) Age-related differences in movement representation. Neuroimage 17: 1720–1728. 12498746 10.1006/nimg.2002.1309

[78] Svensson P, Romaniello A, Arendt-Nielsen L, Sessle BJ (2003) Plasticity in corticomotor control of the human tongue musculature induced by tongue-task training. Exp Brain Res 152: 42–51. 12830348 10.1007/s00221-003-1517-2

[79] Tyc F, Boyadjian A, Devanne H (2005) Motor cortex plasticity induced by extensive training revealed by transcranial magnetic stimulation in human. Eur J Neurosci 21: 259–266. 15654863 10.1111/j.1460-9568.2004.03835.x

[80] Brogardh C, Johansson FW, Nygren F, Sjolund BH (2010) Mode of hand training determines cortical reorganisation: a randomized controlled study in healthy adults. J Rehabil Med 42: 789–794. 20809062 10.2340/16501977-0588

[81] Suzuki M, Kirimoto H, Onishi H, Yamada S, Tamaki H, et al (2012) Reciprocal changes in input-output curves of motor evoked potentials while learning motor skills. Brain Res 1473: 114–123. 22871269 10.1016/j.brainres.2012.07.043

[82] Roricht S, Machetanz J, Irlbacher K, Niehaus L, Biemer E, et al (2001) Reorganization of human motor cortex after hand replantation. Ann Neurol 50: 240–249. 11506408 10.1002/ana.1091

[83] Kouchtir-Devanne N, Capaday C, Cassim F, Derambure P, Devanne H (2012) Task-dependent changes of motor cortical network excitability during precision grip compared to isolated finger contraction. J Neurophysiol 107: 1522–1529. 22157124 10.1152/jn.00786.2011

[84] Zoghi M, Nordstrom MA (2007) Progressive suppression of intracortical inhibition during graded isometric contraction of a hand muscle is not influenced by hand preference. Exp Brain Res 177: 266–274. 16947062 10.1007/s00221-006-0669-2

[85] Goodwill AM, Pearce AJ, Kidgell DJ (2012) Corticomotor plasticity following unilateral strength training. Muscle Nerve 46: 384–393. 22907229 10.1002/mus.23316

[86] Liepert J, Haevernick K, Weiller C, Barzel A (2006) The surround inhibition determines therapy-induced cortical reorganization. Neuroimage 32: 1216–1220. 16809053 10.1016/j.neuroimage.2006.05.028

[87] Butler AJ, Kahn S, Wolf SL, Weiss P (2005) Finger extensor variability in TMS parameters among chronic stroke patients. J Neuroeng Rehabil 2: 10. 15927075 10.1186/1743-0003-2-10 PMC1175099

[88] Corneal SF, Butler AJ, Wolf SL (2005) Intra- and intersubject reliability of abductor pollicis brevis muscle motor map characteristics with transcranial magnetic stimulation. Arch Phys Med Rehabil 86: 1670–1675. 16084825 10.1016/j.apmr.2004.12.039 PMC3575081

